# What is the Role of Internalizing and Externalizing Symptoms in Adolescent Suicide Behaviors?

**DOI:** 10.3390/ijerph16142511

**Published:** 2019-07-14

**Authors:** José Antonio Piqueras, Victoria Soto-Sanz, Jesús Rodríguez-Marín, Carlos García-Oliva

**Affiliations:** Miguel Hernandez University of Elche, 03202 Alicante, Spain

**Keywords:** suicide, adolescence, symptomatology, internalizing, externalizing, longitudinal

## Abstract

Suicide is the second leading cause of death in adolescents and young adults aged 15 to 29 years. Specifically, the presence of internalizing and externalizing symptomatology is related to increased risk for suicide at these ages. Few studies have analyzed the relations between these symptoms and their role as mediators in predicting suicide behavior. This study aimed to examine the relation between internalizing and externalizing symptomatology and suicide behaviors through a longitudinal study. The sample consisted of 238 adolescents aged 12 to 18 years. The data were analyzed via the PROCESS Statistical Package. The main results showed that previous depression symptoms had a significant indirect effect, through previous suicide behaviors and current depression symptoms, on current suicide behaviors, accounting for 61% of the total variance explained. Additionally, being a girl increased this risk. Therefore, the implementation of early identification and intervention programs to address youth symptoms of depression and suicidal behaviors could significantly reduce the risk for future suicidal behaviors in adolescence.

## 1. Introduction

Suicide is a public health problem, and is the second leading cause of death among adolescents and young adults aged 15 to 29 years [1,2]. In Spain, the consummated suicide rate per 100,000 inhabitants in 2017 was 0.19 in children under 15 years of age and 2.23 in the 15–19 years age group [3]. Although more studies are needed on the etiology of suicide, there are well-known factors that predict, to some extent, the risk for suicidal behavior in this age group: previous existence of suicidal behaviors (thoughts or attempts) [4,5,6]; exposure to traumatic stressful events, such as abuse or victimization [7]; existence of legal factors [8,9,10]; association with certain psychological factors, such as hopelessness, impulsivity, and low self-esteem [11,12,13,14,15]; and presence of somatic or disabling problems [16,17,18] or previous history of mental disorders, specifically emotional and behavioral disorders [19,20]. In addition, gender differences have been suggested, given that rates of suicidal ideation and attempts are higher in women [21,22,23].

### Internalizing and Externalizing Symptomatology as a Risk Factor in Adolescents

Overall, the association between suicide and presence of mental disorders is clear, but uncertain when we consider the only presence of symptoms of emotional and behaviors disorders. These symptoms would correspond to internalizing and externalizing behaviors and symptoms, following Achenbach, Edelbrock, & Howell’s [24] classification, with internalizing symptoms entailing manifestations of anxious, depressive, and somatic problems, and externalizing symptoms including problems related to aggressiveness, inattentiveness, disobedience, and criminal behavior. Specifically, the presence of emotional and behavioral disturbances appears as a recurrent predictor in several studies [20,25,26,27,28,29,30,31]. The simultaneous presence of several internalizing problems, particularly anxiety and depression, is associated with an increased risk for suicide attempts [32,33].

However, according to a recent meta-analysis of adolescents and young adults [34], both the presence of these disorders and of symptomatology represent a risk factor for suicidal behaviors, specifically symptoms of anxiety, depression, and legal problems.

During adolescence, levels of anxiety and depression are very high, with percentages of 12.6% and 32.4%, respectively [35]. In the non-clinical population, depressive symptoms alone do not directly predict suicide risk, but there are other additional variables that mediate this association [36]. Further, there is no consensus on the role played by the single presence of symptoms of emotional (depression and anxiety) and behavioral disorders in adolescent suicide behaviors.

This study aimed to examine the relations between internalizing and externalizing symptomatology and suicide behaviors through a longitudinal study in an adolescent population aged from 12 to 18 years. It is expected that the internalizing and externalizing symptomatology at times 1 and 2 will present a positive and significant correlation between them and, in turn, that both will correlate significantly with the suicide behaviors at times 1 and 2 (Hypothesis 1). It is also expected that internalizing and externalizing symptomatology at time 2 and the presence of suicidal behaviors at time 1 will work as mediating variables in the relation between Internalizing Symptomatology at time 1 and Suicidal Behaviors at time 2 (Hypothesis 2). Finally, it is hypothesized that depression symptoms will explain the greatest variance of suicide at time 2 (Hypothesis 3), according to previous studies [20].

## 2. Materials and Methods

### 2.1. Participants

This study falls within the framework of a broader research on mental health in adolescents (2016/2017) (DPS-JPR-004–16) [37], where 909 students between the ages of 12 and 18 years from the province of Alicante formed an initial sample at time 1 (t1). The second time of assessment was made six months later (t2) and consisted of 238 adolescents. The distribution according to gender was 63.86% (152) boys aged 12 to 18 years, with a mean age of 14.85 years (SD = 1.35). The majority of adolescent participants were of Spanish origin (98.68%). This percentage was lower but quite representative of the rate of immigrant adolescents attending school in Spain (8.68%) [38]. In relation to the socioeconomic status (SES), 80% of the sample belongs to middle class.

### 2.2. Variables and Instruments

Information on sociodemographic variables was obtained through an ad-hoc questionnaire. The Family Affluence Scale [39] was used to measure SES. The FAS assesses household purchasing power or family wealth and consists of four questions about the availability of personal rooms, number of household cars and computers, and family vacations taken in the last 12 months. The scale was developed to reliably estimate family SES in (young) children using questions they are likely to know about. It has shown good criterion and construct validity in previous studies [40].

The DetectaWeb-Distress Scale [41,42] was used to obtain information on suicidal behaviors and internalizing symptoms. It consists of 30 items that evaluate major depression, dysthymia, panic/agoraphobia disorder, generalized anxiety disorder, post-traumatic stress disorder, obsessive-compulsive disorder, separation anxiety disorder, social phobia, specific phobia, and suicidal behavior (ideation, plans, and attempts) through a Likert-type response scale (0 = never, 1 = sometimes, 2 = often, and 3 = always). For suicidal information, we used the score in suicidal behavior, made up of three items that ask whether the idea, plan, and/or attempt to commit suicide had presented (score range between 0 and 9) through the items “Have you ever thought about killing yourself?”, “Have you ever thought of a way to kill yourself?” and “Have you ever intent to kill yourself”. For internalizing symptoms, we used the composite score obtained from the addition of depressive (major depression and dysthymia) and anxiety symptoms (sum of panic/agoraphobia disorder, generalized anxiety disorder, separation anxiety disorder, social phobia, and specific phobia subscales). This factor solution was supported by Garcia-Olcina et al. [41]. Higher scores were interpreted as greater levels of suicidal behavior and internalizing symptomatology. The scores for suicide behaviors and specific symptoms subscales have previously shown adequate psychometric properties of reliability (α = 0.87) and validity (*r* = 0.81) [39,40]. For the present study sample, the internal consistency coefficients were good for suicide t1 and t2 (omega = 0.86 and 0.90), depression t1 and t2 (omega = 0.90 and 0.90), anxiety t1 and t2 (omega = 0.85 and 0.87), and the internalizing subscales scores, t1 and t2 (omega = 0.88 and 0.90).

The Strengths and Difficulties Questionnaire (SDQ) [43] was used to evaluate externalizing symptomatology. Previous studies have supported the use of broader externalizing SDQ subscales [40]. Indeed, factor analyses supported second-order internalizing and externalizing factors, and their subscales showed good convergent and discriminant validity across informants, as well as with respect to clinical disorder [44,45]. Consequently, we only administered the conduct problems and hyperactivity-inattention subscales, which then provided the calculation of the externalizing SDQ subscale. It consisted of ten items, with five items for each one. Providing the presence of externalizing symptoms over the last six months with the following answer options for each item being “Not true,” “Somewhat true,” and “Certainly true.” The externalizing SDQ subscale score ranged from 0 to 20 points [44]. The externalizing SDQ subscale showed acceptable psychometric properties among the present population at t1 and t2 (omega = 0.70 and 0.68).

### 2.3. Procedure

This work was carried out with the permission of the Regional Secretariat for Education and Research and the Project Evaluation Agency of the Miguel Hernández University (Reference DPS.JPR.04.16). After obtaining the authorizations, the 11 centers included in the proposal were contacted (convenience sampling), of which seven agreed to participate. Informed consent was requested to be signed by at least one of the parents and the participant. 

For the first time (t1), prior to the collection of information, a session was held with the school teachers where they learned to carry out the administration of the online survey. Data collection was carried out in the computer rooms via a web-based survey, the DetectaWeb platform for the detection of mental health in children and adolescents (see complete protocol in reference 39; for more information, see http://www.detecta-web.com/blog/). Six months later, the procedure was conducted for a second time (t2). The same teachers carried out the data collection.

### 2.4. Data Analyses

Data analyses were carried out using the SPSS (v24) software for Apple [46]. The Kolmogorov-Smirnov test was used to evaluate the normality of the sample, deviations to normality for asymmetry (skewness) by the concentration of data in the mean (kurtosis), and the box diagram for outlier’s analysis. McDonald’s omega coefficients were calculated to estimate the internal consistency of each of the subscales and total scale scores for our sample [47], as this was a more appropriate measure compared with Cronbach’s alpha [48]. Values of internal consistency are considered as acceptable between 0.70 and 0.90 [49], although according to Katz [50], values higher than 0.65 are also accepted. Pearson’s correlations were calculated between all variables to identify possible covariates and to study associations between variables according to the study hypotheses. Descriptive information was obtained in relation to the scores reported by comparing the group of boys versus the group of girls (mean and standard deviations) through the student’s *t*-test for independent samples; effect sizes were calculated (Cohen’s d (d): 0.2 = small, 0.5 = medium, and 0.8 = large) [51]. Simple regression analyses were a priori performed to establish whether or not the relation was significant between the different predictor variables and the criterion variable. Finally, the SPSS computer tool PROCESS [52] was used to analyze conditional processes (model 6). Through this tool, two or more mediators could be tested simultaneously. It also provided an actual test of mediation, including bootstrapping, to quantify the stability of the indirect effect. As the sample was not very large, we applied a non-parametric bootstrap approach to evaluate indirect effects [50]. Thus, if the 95% confidence interval (CI; including the lower and upper limits) does not contain zero, the indirect effects are significant [53].

Regression coefficients were estimated using a model, in which associations were calculated using the bootstrapping procedure (5000 resamples) and results were considered significant when the value was less than 0.05. The statistical approach proposed was a top–down strategy, so that statistical approach was posed from general models where the relation of the general symptomatology was examined internalizing and externalizing with suicide, down to the relation of specific depressive symptoms within internalizing symptoms and with suicide, longitudinally.

## 3. Results

### 3.1. Preliminary and Descriptive Analyses

No data were found to indicate violation of the assumptions of normal variable distribution. Although atypical cases were detected, we decided not to remove them from the sample for reasons of ecological validity. 

The descriptive data of the variables used in this study for the total sample are shown in Table 1. According to the information collected, the mean scores of the girls were higher on all scales, although they differed significantly and showed a large effect size on internalizing symptomatology.

The correlations between the different variables used in this study are shown in Table 2, in which we can see that all the values are positive and significant. Major correlations were observed between the same variables measured at times 1 and 2. 

Overall, at time 1, 46 adolescents scored 1 (sometimes) or more on the suicide scale (19.3%) and at time 2 were 48 adolescents (20.2%). The frequency distribution of the score obtained from Suicide Scale showed is shown in Table 3. The majority of teens who scored more than 1 on the scale indicated “Sometimes” in Plan, Ideation, and less often in Attempt.

### 3.2. Internalizing and Externalizing Symptoms at t1 and t2 in the Prediction of Suicidal Behaviors at t2.

Preliminary analysis of regression showed that Internalizing Symptomatology (t1 and t2), Externalizing Symptomatology (t1 and t2), and Suicidal Behaviors (t1) were individual predictors of Suicidal Behaviors (t2). When they were entered simultaneously in the same model (controlling for the gender) they accounted for 54% of the explained variance of Suicide Behavior. Regression analysis showed that the included variables were statistically significant with Suicidal Behavior (t2), and the highest explained variance of Suicide Behaviors (t2) was given by the Suicide Behaviors variable (t1) (46%). These results suggest that all the constructs are relevant in determining adolescents’ Suicidal Behaviors (t2). To further explore this hypothesis, we built a mediation model to test the relationship between these variables.

To obtain a global representation of the relations between Internalizing and Externalizing Symptomatology on the prediction of current Suicidal Behaviors (t2), a mediation model was proposed. In this model, Internalizing Symptomatology t1 was the predictor variable; Internalizing Symptomatology t2, Externalizing Symptomatology t1 and t2, and Suicide Behaviors t1 were the mediating variables; and current Suicide Behaviors (t2) was the outcome. Given the gender differences in internalizing symptoms, we decided to add gender as a covariate.

**Figure 1:**
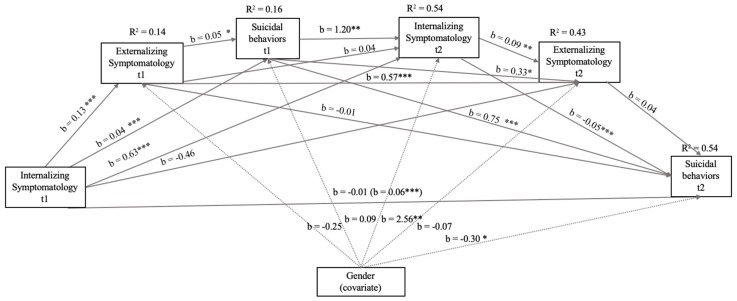
Model of mediation of the relations between Internalizing Symptomatology (t1) through Internalizing Symptomatology (t2), Suicide Behaviors (t1 and t2), Externalizing Symptomatology (t1 and t2), and Suicide Behaviors (t1) with Suicide Behaviors (t2) after controlling for gender (girl). In this statistical diagram, values are represented with non-standardized regression coefficients. In the association between Internalizing Symptomatology t1 and Suicide Behaviors t2, the value outside parentheses represents the direct effect, and the value in parentheses represents the total effect of the bootstrapping analysis of Internalizing Symptomatology t1 on Suicide Behaviors t2 after the inclusion of mediation variables. *** *p* < 0.001, ** *p* < 0.01, * *p* < 0.05.

The correlation coefficients in the different relations are presented in Figure 1. In this model, the correlations with greater significance (*p* < 0.001) and higher b values were those between the same variables measured at time 1 and at time 2, as well as the influence of Suicidal Behaviors (t1) on Internalizing (t2) and Internalizing Symptomatology (t1) on Externalizing Symptomatology (t1). The total model explained 54% of the total variance of current Suicide Behaviors. The total effect was b = 0.06 (Standard Error [EE] = 0.01, t = 5.65, *p* < 0.001, 95% CI: 0.04/0.07). In terms of mediating variables, neither Externalizing Symptomatology t1 nor t2 was significantly associated with Suicide Behaviors t2, although Externalizing Symptomatology t1 was significantly associated with Suicide Behaviors t1. The covariate included (being a girl) was related to Internalizing Symptomatology and Suicide Behaviors t2.

**Figure 2:**
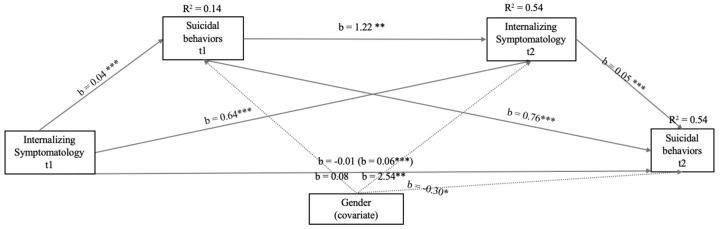
Model of mediation of the relations between Internalizing Symptomatology (t1 and t2) and Suicide Behaviors (t1) with Suicide Behaviors (t2) after controlling for gender (girl). In this statistical diagram, values are represented with non-standardized regression coefficients. In the association between Internalizing Symptomatology t1 and Suicide Behaviors t2, the value outside parentheses represents the direct effect, and the value in parentheses represents the total effect of the bootstrapping analysis of Internalizing Symptomatology t1 on Suicide Behaviors t2 after the inclusion of the mediation variables. *** *p* < 0.001, ** *p* < 0.01, * *p* < 0.05.

Given the low predictive capacity of externalizing symptomatology in the model, we created a model omitting these variables (see Figure 2). In this model, the correlations with greater significance (*p* < 0.001) and higher b values were between Suicidal Behaviors (t1) and Internalizing Symptomatology (t1. There were also significant associations between the same variables measured at time 1 and at time 2 (*p* < 0.001). The total model accounted for 54% of the total variance explained of Suicide Behaviors t2, with the total effect of Internalizing Symptomatology t1 being positive and significant (b = 0.06, EE = 0.01, t = 5.65, *p* < 0.001, 95% CI: 0.04/0.07). Similarly, the effect of the covariate was the same as in the previous model (Figure 1): positive and significant.

**Figure 3:**
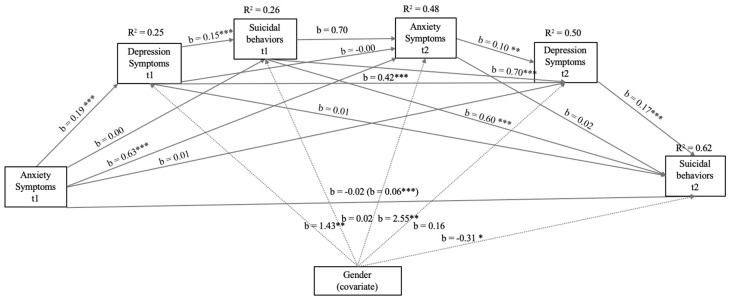
Model of mediation of the relations between Symptoms of Anxiety (t1 and t2), Symptoms of Depression (t1 and t2), and Suicide Behaviors (t1) with Suicide Behaviors (t2) after controlling for gender (girl). In this statistical diagram, values are represented with non-standardized regression coefficients. In the association between Anxiety Symptoms t1 and Suicide Behaviors t2, the value outside parentheses represents the direct effect and the value in parentheses represents the total effect of the bootstrapping analysis of Anxiety Symptoms t1 on Suicide Behaviors t2 after the inclusion of the mediating variables. *** *p* < 0.001, ** *p* < 0.01, * *p* < 0.05.

As can be seen in Figure 3, the new model was created to analyze those internalizing symptoms that predicted, to a greater extent, Suicidal Behaviors t2. In this model, the correlations with greater significance (*p* <.001) and higher b values were between the same variables measured at time 1 and time 2. The effect of Anxiety Symptoms (t1) on Depression Symptoms (t1), as well as of Depression Symptoms (t1) on Suicidal Behaviors (t1) were also significant. In this model, the explained variance of Suicide Behaviors t2 increased to 62%, with a total effect between Anxiety Symptoms t1 on Suicide Behaviors t2 and the remaining variables mediating b = 0.06 (EE = 0.01, t = 5.65, *p* < 0.001, 95% CI: 0.04/0.07). However, Anxiety Symptoms had no association with or significance to Suicide Behaviors, although Anxiety Symptoms t1 was significantly associated with Suicide Behaviors t1. In the case of the included covariate, being a girl was significantly associated with Depression Symptoms t1, Anxiety Symptoms t2, and Suicide Behaviors t2. 

**Figure 4:**
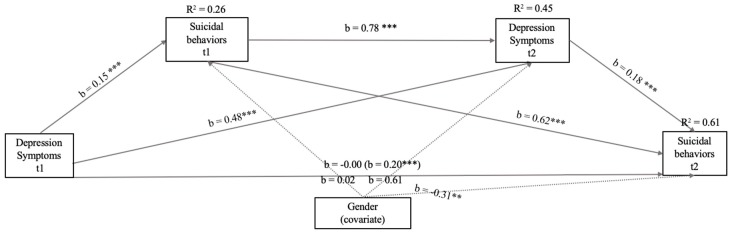
Model of mediation of the relations between Symptoms of Depression (t1 and t2) and Suicide Behaviors (t1) with Suicide Behaviors (t2) after controlling for gender (girl). In this statistical diagram, values are represented with non-standardized regression coefficients. In the association between Depression Symptoms t1 and Suicide Behaviors t2, the value outside parentheses represents the direct effect and the value in parentheses represents the total effect of the bootstrapping analysis of Depression Symptoms t1 on Suicide Behaviors t2 after the inclusion of the mediation variables. *** *p* < 0.001 ** *p* < 0.001 * *p* < 0.05.

In view of the fact that Anxiety Symptoms were not significantly related within the model to Suicide Behaviors t2, we decided to create a final model including only depression symptoms at different times (see Figure 4). In this model, the correlations with greater significance (*p* < 0.001) and higher b values were between the same variables measured at time 1 and time 2 (*p* < 0.001). The relationship between Depression Symptoms (t1) and Suicidal Behaviors (t1) was also significant, but with a lower b value.

In including only Suicide Behaviors t1 and having Depression Symptoms t2 mediate the relation between Depression Symptoms t1 and Suicide Behaviors t2, the model explained 61% of the total variance for Suicide Behaviors t2, with being a girl increasing the risk for Suicide Behaviors t2. This model showed total mediation with b = 0.20 (EE = 0.02, t = 8.52, *p* < 0.001, 95% CI: 0.15/0.24).

## 4. Discussion

Considering the high prevalence of suicide behaviors among adolescents and their correlation with internalizing and externalizing symptoms, the aim of the present study was to confirm the relation between internalizing and externalizing symptoms and suicide behaviors through a longitudinal study in an adolescent population, including gender as a covariate.

To determine which variables had a mediating role, we constructed a first model with all variables included. According to the results of this study, and with respect to hypothesis 1, the presence of internalizing and externalizing symptoms and suicidal behaviors were predictors of the same symptoms longitudinally, although only suicidal behavior was a moderate predictor of suicide over time. In this model, the presence of current internalizing symptomatology and previous suicide behaviors mediated between having previously presented internalizing symptomatology and presenting current suicide behaviors. This correlation confirms a previous report [54]: in a three-factor model, suicidal tendency (measured with suicidal ideation and suicide attempts across life), internalizing disorders (assessed with lifetime diagnoses of major depressive episodes and post-traumatic stress disorders), and externalizing disorders (indicated by lifetime diagnoses of conduct disorder, alcohol abuse, and drug abuse) were positively interrelated.

Hypothesis 2 was not strictly fulfilled, although the predictive role of internalizing symptoms on suicidal behaviors was confirmed. We found a mediating role for Suicidal Behaviors t1 and Internalizing Symptomatology t2 in this relation. Namely, emotional symptoms could predict suicide longitudinally as long as these symptoms were associated with suicidal behaviors, and when internalizing symptoms persisted over time. A related study reported a significant relation between suicide and internalizing disorders, but not with externalizing disorders [55]. However, in another study with Asian patients who were admitted to the emergency department, as compared to adults, adolescents encountered comparatively fewer external problems including financial problems and resulted in reduction of externalized symptoms [56]. This fact may be because, in previous studies, affective disorders in general and depression comorbid with conduct problems in particular, have been found to increase the risk for suicide among adolescents [57,58]. Therefore, the most potent relationship may be when both comorbid internalizing symptoms and externalizing symptoms act as a predictor.

Finally, regarding the role of depression symptoms in suicidal behavior, hypothesis 3 was confirmed. Although Figure 3 shows that anxiety symptoms are related to depression, they are not significantly related to Suicide Behaviors t2. This relation between anxiety and depression was expected; anxious young people have more difficulty relating to others (e.g., deficits in social skills and conflict resolution [59,60]). It is logical that anxiety can predict future depression or depression symptoms, as during adolescence, social relationships and peer support are essential to prevent the onset of depression problems [61]. However, the literature links depression more closely to suicidal behaviors [62]. In a meta-analysis [20], depressive disorder is found to be the mental disorder more strongly associated with increased risk for suicide.

In addition, as displayed by the different models, and as expected, we found statistically significant differences between boys and girls. This evidence was already collected in a previous meta-analysis [63] that concluded differences in gender and suicidal behavior, with an augmented risk for women who present depressive symptoms. This association with gender has also been seen in previous studies. According to the literature, men have a higher suicide mortality rate worldwide, and rates of non-fatal suicidal behaviors are higher in women. Gender, therefore, is one of the most studied predictors [64]. Indeed, this phenomenon has been called the "gender paradox" [65]. These differences are even also found in adolescence, as girls tend to internalize suffering and boys to externalize it [66].

## 5. Conclusions

The main result of this study was that depressive symptoms longitudinally predict suicidal behavior in adolescents, although this relation occurs if depression and suicidal behaviors previously occurred at the same time, and depression symptoms persisted longitudinally (t2). In other words, depression predicted suicide when it was associated with the presence of suicidal behaviors at the same time and these depressive symptoms persisted in the long term. Additionally, this relation was more pronounced among girls. 

One of the main contributions of this study its focus on the interaction between internalizing and externalizing symptoms during adolescence, compared with the literature examining this relation in adult population.

However, certain limitations must be considered. Despite being a longitudinal study, the sample was small, belonging only to one of the 52 provinces of Spain. Consequently, a larger sample from different regions of the country would provide more widely generalizable results. It is important to emphasize that it was difficult to gather a sample of adolescents to participate in a longitudinal study in which we asked about suicide. Additionally, the sample size was too small to the potential differences in the relationship between internalizing symptoms and specific suicidal behaviors, such as ideation, plan, or attempt.

It should be noted that the instruments used were self-reported measures. The results would be more representative and of greater relevance if structured clinical interviews with clinical samples could be carried out. Additionally, concerning the selected instruments to assess internalizing and externalizing symptoms, contrary to what might be expected, we did not include the internalizing SDQ scale. The reason was it only provides scores of emotional and peers relationship problems, and not of specific symptoms of anxiety and depression, which are central in internalizing problems and potentially have differential weight in their relation with suicide. Consequently, we opted for DetectaWeb-Distress, a new instrument that provides both specific symptoms of anxiety and depression and a composite score of internalizing symptoms. 

Another limitation is that this study did not measure the relationship between borderline personality disorder and suicide in adolescents. Clinically, adolescents suffering from borderline personality disorder often attempt suicide. Borderline personality disorder is characterized by affect dysregulation, self-disturbances, and behavioral and interpersonal dysregulation [67]. Borderline personality disorder has both internalizing and externalizing symptoms [68]. Further study is required to study the relationship between borderline personality disorder, internalizing and externalizing symptoms and suicide.

In conclusion, through a mediation analysis approach, we established that the presence of symptoms of depression in the last six months may be a risk factor for current suicide behaviors among adolescents, but especially when they were accompanied by suicide behaviors, and additionally when depression was maintained or repeated six months later. This risk or association was greater for adolescent girls. 

It should be mentioned that not only are adolescents with disorders at risk, but the fact that they present high levels of non-clinical depressive symptoms and suicidal behaviors may be indicative of alertness. The governments should invest in measures that provide early detection; interventions for symptoms of depression and suicidal behaviors could significantly reduce the risk for future suicidal behaviors in adolescents.

## Figures and Tables

**Table 1:** Mean (M), standard deviation (SD), differences between boys and girls, student’s t-test (t), effect size (d) (N = 240).

Variables	M Boys (*N* = 152)	*SD*	*M* Girls (*N* = 86)	*SD*	*t*	*d*
Suicide Behaviors, t1	0.29	0.77	0.65	1.29	−2.41 **	0.33
Suicide Behaviors, t2	0.42	1.23	0.68	1.37	−1.59	0.20
Internalizing Symptomatology, t1	15.8	7.37	22.63	8.99	−6.29 ***	0.83
Internalizing Symptomatology, t2	13.83	8.48	21.16	7.99	−6.65 ***	0.88
Externalizing symptomatology, t1	5.57	3.07	6.20	2.53	−1.63	0.22
Externalizing symptomatology, t2	5.84	3.15	6.61	2.67	−1.90	0.26

Note. *** *p* < 0.001, ** *p* < 0.01.

**Table 2:** Bivariate correlations between variables.

Variables	1	2	3	4	5	6
(1) Suicide Behaviors, t1	-					
(2) Suicide Behaviors, t2	0.68 ***	-				
(3) Internalizing Symptomatology, t1	0.38 ***	0.35 ***	-			
(4) Internalizing Symptomatology, t2	0.39 ***	0.48 ***	0.71 ***	-		
(5) Externalizing Symptomatology, t1	0.25 ***	0.22 ***	0.37 ***	0.29 ***	-	
(6) Externalizing Symptomatology, t2	0.31 ***	0.33 ***	0.30 ***	0.37 ***	0.61 ***	-

Note. *** *p* < 0.001.

**Table 3:** Frequency Distribution of the score of Suicide Scale.

	Ideaction t1 N (%)	Ideation t2 N (%)	Plan t1 N (%)	Plan t2 N (%)	Attempt t1 N (%)	Attempt t2 N (%)
Never (score 0)	204 (85.7)	201 (84.5)	199 (83.6)	197 (82.8)	226 (95.0)	221 (92.9)
Sometimes (score 1)	29 (12.2)	28 (11.8)	31 (13.0)	29 (12.2)	12 (5.0)	14 (5.9)
Many times (score 2)	4 (1.7)	8 (3.4)	8 (3.4)	10 (4.2)	-	2 (0.8)
Always (score 3)	1 (0.4)	1 (0.4)	-	2 (0.8)	-	1 (0.4)
Total (score 1–3)	34 (14.3)	37 (15.5)	39 (16.4)	41 (17.2)	12 (5.0)	17 (7.1)
